# Positive time use: a missing link between time perspective, time management, and well-being

**DOI:** 10.3389/fpsyg.2024.1087932

**Published:** 2024-02-26

**Authors:** Evgeny Osin, Ilona Boniwell

**Affiliations:** ^1^LINP2, University of Paris Nanterre, Nanterre, France; ^2^International Laboratory of Positive Psychology of Personality and Motivation, HSE University, Moscow, Russia; ^3^School of Psychology, University of East London, London, United Kingdom; ^4^Positran, Épone, France

**Keywords:** balanced time perspective, Zimbardo Time Perspective Inventory, subjective well-being, temporal well-being, time management

## Abstract

**Introduction:**

The notion of satisfaction with the use of one’s time has not been operationalized previously. Based on qualitative interviews, we propose a concept of positive time use comprised by four components: self-congruence of daily activities, balance between activities, efficient use of time, and a sense of mastery over one’s time.

**Methods:**

Using data from two UK adult samples (*N* = 173 and *N* = 357), we developed a new measure, Positive Time Use Inventory (PTUI), and investigated its structural and convergent validity.

**Results and discussion:**

The associations of positive time use with balanced time perspective, affect balance, satisfaction with life, sense of coherence, and self-reported satisfaction with time use indicate convergent and discriminant validity of the new measure. Positive time use partially explained the associations of balanced time perspective with subjective well-being and fully mediated the effects of future time perspective and time management on subjective well-being. We propose positive time use as a new model of temporal well-being, which brings together the notions of work-life balance, time efficiency, and time mastery in a single comprehensive framework, helping to inform the time management coaching interventions.

## Introduction

The twenty-first century has been marked by a renewed interest in human well-being and the development of positive psychology, a scientific study of the things that make life worth living. However, within this rapidly growing field, there has been very little work focused on the positive aspects of our relationship with time, one of the fundamental conditions of human existence.

In this paper, we propose a positive psychological model of time use informed by existing theory and by qualitative studies of time use satisfaction. The model describes four phenomenological criteria of positive use of time that entails a combination of efficiency and fulfillment in one’s daily life: self-congruence and balance of activities, as well as efficiency and a sense of mastery with respect to time. We also present two empirical studies aimed to develop and refine a self-report measure of positive time use that could benefit researchers and practitioners interested in exploring the humans’ relationship with time.

### The problem of time pressure

Time is a basic dimension of our lives: we live in a world of time, where the present evolves from the past and becomes the future (Boss, 1963). Whether we are aware of it or not, time keeps passing; after all, life is only the amount of time each of us has at our disposal. Even though the very passage of time is outside our control, we normally have a choice concerning the activities, people, and things that we devote our time to. Time appears as a limited resource that we continuously consciously or unconsciously distribute between the numerous activities that comprise our life and compete for our attention. Like money, time has a zero-sum character (Robinson, 2002): by spending it on one thing we inevitably spend less time on another, and we continuously face the experience of not having enough time (Mogilner et al., 2018).

Concepts such as “time famine” (Banks, 1983; Perlow, 1999), “time crunch” (Robinson and Godbey, 1997), “time poverty” (DeGraaf, 2003), and “time pressure” (Goodin et al., 2005) have become all too familiar. Chronic time pressure has been recognized as an “unavoidable experience of daily life in highly industrialized societies” (Szollos, 2009, 345) and conceptualized as a combination of an objective time shortage and a subjective experience of being rushed. Despite the advances in technology that enable us to do things faster and to multitask, thus using our time more “efficiently” in terms of how much we manage to accomplish in an average day, people’s subjective experience of time pressure does not seem to improve (Newport, 2015). Although the objective amount of work hours has declined over the past century, paradoxically, people nowadays feel they have *less* time to spare (Robinson and Godbey, 1997; Pentland et al., 1999; Sullivan and Gershuny, 2001). This may be explained by the time use choices people make and the extent to which they feel in control of their time (Goodin et al., 2005, 2008).

Time management programs aimed at optimizing one’s time use have gained popularity in recent decades. The limited existing research evidence suggests that although these programs may increase participants’ subjective feelings of control over their time and relieve time pressure and stress, the evidence concerning their impact on participants’ actual performance and well-being is mixed, at best (Claessens et al., 2007; Aeon and Aguinis, 2017). Indeed, while intensive time management may free up some time by squeezing an extra hour or two out of each day, it may also result in “time deepening,” cramming a larger number of activities into a shorter amount of time, leading to negative experiences of time fragmentation, time strain, and being rushed (Robinson and Godbey, 1997).

And having more time to spare does not always make people happier either. Despite an objective trend reflecting an increase in leisure time between 1965 and 2005, the proportion of time Americans spent doing pleasant vs. unpleasant activities has remained nearly the same (Krueger et al., 2009). Even though weekly workload and the amount of free time do predict time pressure and feeling stressed, their associations with satisfaction with the use of time are quite weak (Roberts, 2007; Zuzanek, 2017), suggesting that people may be satisfied with their use of time even when they have very little time to spare. Though recent findings show that people who spend money to buy more time do indeed experience greater satisfaction (Whillans et al., 2017), the reason is probably related to why people value time and what they use it for (Whillans and Dunn, 2019).

It seems that the time crunch problem cannot be solved by simply increasing the amount of time available to individuals or by giving them tools to manage it more efficiently. It is not having *more* time, but having more *satisfying* time that people seem to need. But what is positive time use and what it is that helps people to experience time as spent well?

### Having a “good” time vs. using one’s time well

One way to answer the question of a “good” time is to equate it with pleasant time. Within this paradigm, “good” time use can be defined as spending time in activities that bring positive momentary emotional experiences or receive a retrospective positive evaluation. This approach can be labeled hedonic and it has laid the ground for a number of well-known time use studies relying on subjective indicators, such as the U-Index (Kahneman and Krueger, 2006; Krueger et al., 2009). However, eudaimonic well-being research shows that experiences bringing momentary pleasure may not necessarily be satisfying in the long term (Vittersø, 2013, 2016; Waterman, 2013).

From the time management point of view, “good” time use can be defined as efficient time use, that is, objectively, absence of procrastination and time-wasting, and, subjectively, a sense of control over one’s time and absence of time poverty and time stress, having “enough” time. Research shows that the experience of time scarcity or time poverty is associated with engaging in inefficient time management behaviors (Kaufman-Scarborough and Lindquist, 2003) and also with lower subjective well-being (Kasser and Sheldon, 2009). However, the subjective notion of having “enough” time can hardly be defined without specifying *what* it is that the time is needed for and *why*.

Both of these approaches to defining positive time use (as either “satisfying” or “efficient”) seem incomplete. People who typically spend time on enjoyable activities may fail to achieve important life goals and face disappointment in the long term. On the other hand, people who are highly efficient in their use of time may eventually face burnout and depression in case their daily activities fail to bring them lasting fulfillment (Längle, 2003). Curiously, time management literature, with few exceptions (Black and Bailey, 2006), has remained silent about the necessity to like (at least, to some extent) the activities that a person aims to fit into their day. We believe that the definition of positive time use needs to combine and transcend these two, rather simplistic, perspectives, by addressing the question of when and why do activities bring lasting satisfaction.

The answer is provided by the theory of basic psychological needs, a part of Self-Determination Theory (SDT) postulating that satisfaction of the needs for autonomy, competence, and relatedness is essential for positive human functioning (Ryan and Deci, 2017). Within the time use field, Kasser (2009) has introduced the concept of time affluence, an opposite of time poverty, defined as having enough time for activities and experiences that sustain psychological well-being by satisfying the basic psychological needs described in SDT. Christiansen and Matuska proposed a theory of a balanced lifestyle, viewing it as one that helps to meet psychological needs (including, but not limited to the needs for autonomy, competence, and relatedness described in SDT, as well as needs for self-esteem, security, purpose, and health) and is satisfying, healthful, meaningful, and sustainable (Christiansen and Matuska, 2006; Matuska and Christiansen, 2008). Matuska (2010, 2012a,b) operationalized life balance as congruence between desired and actual time spent in activities and equivalence of the degree of discrepancy between the two across various life domains.

Sheldon et al. (2010) provide an extensive critique of this model and propose a two-pronged definition of optimal life balance, viewing it as a distribution of time across activities in various life domains that is objectively equitable (close to uniform) and is also subjectively congruent with one’s ideal time-use profile. In a series of empirical studies using cross-sectional, longitudinal, and experimental designs in two cultures, they found that although the indices operationalizing the subjective and objective balance were only weakly correlated, both predicted well-being and this effect was mediated by the satisfaction of SDT’s basic psychological needs.

Recently, based on a combined body of empirical findings, Sheldon and colleagues have proposed the Eudaimonic Activity Model (EAM) (Sheldon, 2013; Martela and Sheldon, 2019; Sheldon and Lyubomirsky, 2021). According to the EAM, basic psychological need satisfaction (“feeling well”) is an outcome of eudaimonic activities (“doing well”) directed at goals that fit the person and are experienced as personally meaningful, valuable, and concordant with the self. Based on these ideas, positive time use can be defined as using time efficiently and distributing it in a balanced way across activities directed at personally meaningful goals that satisfy basic psychological needs and bring a lasting sense of fulfillment.

### The model of positive time use: a conceptual framework

A first attempt to define “good” time use was made by the second author (Boniwell, 2005, 2006) in her qualitative interview study of time use satisfaction and well-being. She interviewed 22 participants, half of whom were largely satisfied and another half largely dissatisfied with their use of time. Using Interpretative Phenomenological Analysis, she identified 148 emergent themes grouped into 10 general clusters describing a satisfying use of time (ordered in terms of decreasing frequency): balance and variety of activities, discipline and adaptability, achievement vs. wasting time, taking responsibility and feeling in control, prioritization and acceptance of limits, time anxiety and perspectives on time, liking what one does, life goals and worthwhile activities, time management mechanics, and, finally, reflection and evaluation.

These clusters formed four super-ordinate themes, or facets of perceived time use: Motivation (liking what one does in life and perceiving it as worthwhile), Organization (prioritization resulting in a balance of activities and in having time for oneself), Execution (discipline and responsibility, achieving instead of wasting time), and Evaluation (absence of anxiety and lack of control with respect to time). Based on these phenomenological findings, the second author developed a pool of 69 items tapping into satisfaction with time use and conducted an exploratory factor analysis to investigate its structure, discovering four distinct factors corresponding to the proposed facets (Boniwell, 2005, 2006). However, the study was limited with respect to both sample and analytic methods.

Building on these early results and on later theorizing, we propose to define positive time use as a multifaceted construct reflected in four phenomenological experiential indicators:

1. *Self-congruence* of daily activities: an alignment of the things one typically does in everyday life with one’s values, goals, and priorities which results in one’s daily activities being experienced as personally meaningful, important, satisfying, and fulfilling (even if not always pleasant). According to Self-Determination Theory, this experience is an outcome of pursuing goals that satisfy one’s basic psychological needs (Ryan and Deci, 2017, 253).
2. *Balance* between daily activities directed at different goals and life domains. This notion is more general than work-life or work-family balance and refers to a general feeling of satisfaction with the way one distributes one’s time and resources (attention, effort) between the various activities comprising one’s life.
3. *Efficiency* in the use of time: absence of procrastination and of time experienced as wasted in one’s daily life; efficient organization, initiation, and execution of one’s activities.
4. *Control* over one’s time or time mastery: absence of time anxiety and time pressure a daily basis, a sense of being “in control” of one’s time, rather than being overwhelmed and guilty.

In short, we define positive time use as both satisfying (due to having a healthy balance between life areas and choosing self-congruent activities on a daily basis) and efficient (both objectively, spending time efficiently, and subjectively, experiencing a sense of mastery over one’s time). Empirically, we expect that these four components or indicators of positive time use are supposed to form a single second-order dimension (Hypothesis 1).

The construct of positive time use overlaps with earlier constructs, such as time affluence (Kasser, 2009), lifestyle balance (Matuska, 2010), and purpose (Martela and Steger, 2016), but it also brings in the notion of efficient time use developed within time management literature (Bond and Feather, 1988). We believe that it may help to fill the gaps and explain the links between some well-studied individual-difference constructs, such as time perspective and time management, on the one hand, and general subjective well-being, on the other hand. In the following sections, we will discuss the antecedents of positive time use.

### Antecedents of positive time use: balanced time perspective

Time perspective is an individual’s cognitive way of relating to his/her psychological past, present, and future which affects decision-making and subsequent actions (Zimbardo and Boyd, 1999). Empirical studies have shown that time perspective has a powerful influence on almost all domains of human life and is associated with diverse outcomes, such as educational attainment, somatic and mental health, delinquency and substance abuse, sleep and dreaming patterns, choice of romantic partner, economic behavior, sustainable lifestyle, to name only a few (Stolarski et al., 2015a).

A widely adopted empirical model of time perspective was proposed by Zimbardo and is operationalized in the Zimbardo Time Perspective Inventory (ZTPI) (Zimbardo and Boyd, 1999), which measures five distinct dimensions of one’s subjective representation of time: *Past-Negative* (a focus on past experiences that were aversive, noxious, traumatic, or filled with regret), *Past-Positive* (a pleasurable, usually sentimental and nostalgic views of one’s past), *Present-Hedonistic* (living in the present moment, enjoying high-intensity activities, sensation-seeking), *Present-Fatalistic* (helpless and hopeless attitude toward the future in the face of uncontrollable present), and *Future* (orientation toward setting and attaining future goals at the expense of present enjoyment, delaying gratification, considering the consequences of one’s actions and decisions). The validity of the ZTPI as a research tool has been confirmed in numerous studies using various behavioral outcomes, such as substance use, risky driving, health risk taking, etc. (Zimbardo and Boyd, 1999; Boniwell and Zimbardo, 2004, 2015). The ZTPI has been translated into more than 25 languages and validated in at least 33 countries (Sircova et al., 2014, 2015).

Which time perspective is most conducive to well-being? Studies using the ZTPI consistently reveal that individual dimensions of the ZTPI only show weak associations with trait well-being indicators (Boniwell et al., 2010). To address this issue, Zimbardo has proposed the concept of balanced time perspective (BTP), in which “the past, present and future components blend and flexibly engage, depending on a situation’s demands and our needs and values” (Zimbardo, 2002, 62). This notion is based on the idea that an excessive orientation toward any single temporal locus (the past, the present or the future) may be detrimental to well-being.

BTP was defined as a combination of high scores on Past-Positive, Present-Hedonistic, and Future dimensions with low scores on Past-Negative and Present-Fatalistic. Studies using various operationalizations of BTP based on the ZTPI have consistently found that BTP is a better predictor of well-being (life satisfaction, affect balance) and positive functioning (optimism, purpose in life), compared to each of the individual time orientations that comprise it (Drake et al., 2008; Boniwell et al., 2010; Zhang et al., 2013; Stolarski et al., 2015b).

However, despite the well-confirmed associations of balanced time perspective with well-being, the mechanisms underlying these associations are not quite clear. Are people with a balanced time perspective happier because they perceive past and present in a generally more positive way, or are they happier because they have adopted more flexible and optimal strategies of managing their time and manage to achieve a more optimal time use?

Based on the Positive Time Use model, we propose that individuals with a balanced time perspective are happier because they are likely to choose more satisfying activities and to use their time more efficiently. In short, we expect the associations between balanced time perspective and well-being to be mediated by positive time use (Hypothesis 2).

### Antecedents of positive time use: time management behaviors

The issues of time management have enjoyed limited research attention in psychology. Despite the popular appeal of this concept reflected in thousands of self-help books, a 2007 review (Claessens et al., 2007) has only found 35 previous scientific studies, most of which have used one of the three most popular instruments, the Time Management Behavior Scale (TMBS) (Macan et al., 1990), Time Structure Questionnaire (TSQ) (Bond and Feather, 1988), or the Time Management Questionnaire (TMQ) (Britton and Tesser, 1991). Studies using these measures have shown that individuals with higher self-reported time management skills and behaviors tend to estimate the expected time durations more accurately, spend more time on high priority tasks, and are more successful in the academic domain. Time management is positively associated with perceived control of time, job satisfaction, and has a negative relation with job-induced strain and distress; however, its links with job performance were modest, at best (Claessens et al., 2007, 2009; Eerde, 2015).

Despite having a range of empirical instruments, the time management field still lacks theory that would describe how and explain why and whether time management works (Claessens et al., 2007). Macan (1994) suggested that the relationship of time management skills (setting goals and priorities, mechanics of time management, and preference for organization) with their respective outcomes is mediated by perceived control of time. More recent work has pointed out other variables that may play a role, such as team-level effects (Mohammed and Harrison, 2013; Gevers et al., 2016). However, there are still important gaps in the literature concerning the personality and motivational antecedents of time management behaviors and its connection to more general well-being outcomes, outside the professional or academic domains (Claessens et al., 2009).

We attempt to contribute to filling these gaps by studying the associations of self-reported time management behaviors, on the one hand, with time perspective, which may explain the individual differences in adoption of time management behaviors and, on the other hand, with positive time use, which may be facilitated by time management practices and explain their effects on well-being.

The Positive Time Use model allows to explore why and how time management works: we expect that time management is only conducive to well-being when it enables people not only to use their time more efficiently, but also to maintain a better balance in life by devoting more time to activities perceived as important *and* satisfying. In other words, we expect that the associations between time management behaviors and well-being are mediated by positive time use (Hypothesis 3a). At the same time, time management behaviors may be a mechanism underlying the effects of time perspective on positive time use. We expect that time management behaviors mediate the associations of time perspective with positive time use (Hypothesis 3b).

**Figure 1:**
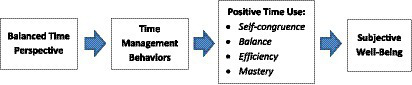
A theoretical model of the associations of positive time use with other constructs.

A summary of the theoretical model is shown in Figure 1. Below we present two studies aiming to validate a measure of positive time use as a single multifaceted construct. The second study also aims to test the hypotheses 2 and 3 by investigating the associations of positive time use and time management behaviors with their hypothesized predictors and outcomes and testing the corresponding mediation models.

## Study 1: development of the positive time use inventory

### Aim

The aim of Study 1 was to explore the structure of the Positive Time Use Inventory and to obtain preliminary evidence of its convergent and discriminant validity against other well-being measures.

### Method

#### Participants and procedure

The sample (*N* = 173) was comprised by part-time university students from the UK, 114 female and 59 male, aged 23 to 85 (*M* = 42.37, *SD* = 12.37), 76.3% of whom were employed or self-employed. The study was approved by the university student research project panel. There were 28 missing responses in the dataset, EM imputation was used to recover the missing data.

#### Instruments

##### Positive Time Use Inventory

The initial pool of 69 items was based on the qualitative interview study of satisfaction with time use undertaken by the second author (Boniwell, 2005, 2006). The items reflected the themes identified using Interpretative Phenomenological Analysis from the interviews of 22 respondents. Based on exploratory factor analyses, 26 items with low communalities (*h* < 0.30) were selected out by the second author (Boniwell, 2005, 2006). The present study used the remaining set of 43 items that the participants evaluated on a 5-point Likert-type response scale from 1 (Strongly disagree) to 5 (Strongly agree).

##### Sense of Coherence Scale (SOC)

The 13-item short version of SOC (Antonovsky, 1987) includes five Comprehensibility items, four Manageability items, and four Meaningfulness items. Each item is evaluated using a 7-point response scale. Sample item: “Do you have the feeling that you do not really care about what goes on around you? 1 = Very seldom or never … 7 = Very often.”

##### Satisfaction with Life Scale (SWLS)

The SWLS (Diener et al., 1985) is a 5-item measure tapping into overall satisfaction with one’s life (sample item: “In most ways my life is close to my ideal”). The SWLS uses a 7-point Likert response scale from 1 (Strongly disagree) to 7 (Strongly agree).

##### Positive and Negative Affect Schedule (PANAS)

The PANAS (Watson et al., 1988) is a list of 20 emotional adjectives (sample items: “interested,” “ashamed”) grouped into two scales, Positive Affect (PA) and Negative Affect (NA). For each adjective, the participants were asked to indicate the extent to which they have felt this way during the past few weeks on a 5-point Likert response scale from 1 (Very slightly or not at all) to 5 (Extremely).

##### Satisfaction with time use

A single item with high face validity (“Overall, I am satisfied with the way I use my time”) rated on a 5-point Likert scale from 1 (Strongly disagree) to 5 (Strongly agree) was used to check the convergent validity of the measure.

The descriptive statistics and reliability coefficients for all the instruments used in this study are presented in Table 1.

**Table 1:** Pearson correlations of PTUI with well-being measures (*N* = 173).

	PTUI	SC	BA	MA	EF	SWLS	PA	NA	SOC
SC	0.76***								
BA	0.78***	0.45***							
MA	0.79***	0.36***	0.51***						
EF	0.50***	0.29***	0.12	0.36***					
SWLS	0.61***	0.59***	0.44***	0.42***	0.26**				
PA	0.46***	0.50***	0.30***	0.25**	0.26**	0.40***			
NA	−0.49***	−0.36***	−0.33***	−0.45***	−0.23**	−0.37***	−0.15		
SOC	0.68***	0.66***	0.44***	0.47***	0.36***	0.64***	0.46***	−0.60***	
SWTU	0.58***	0.39***	0.43***	0.48***	0.39***	0.38***	0.28***	−0.26***	0.33***
M	3.43	3.75	3.34	3.04	3.65	4.38	3.61	2.04	4.65
SD	0.55	0.67	0.77	0.77	0.80	1.45	0.69	0.74	0.96
α	0.88	0.82	0.86	0.78	0.69	0.90	0.88	0.88	0.87

PTUI, Positive Time Use Inventory total score; SC, Self-Congruence; BA, Balance; MA, Mastery; EF, Efficiency; SWLS, Satisfaction with Life Scale; PA, Positive Affect; NA, Negative Affect; SOC, Sense of Coherence. Scale scores are given as item averages. ***p < 0.001, **p < 0.01, *p < 0.05.

### Results and discussion

#### Structure of the Positive Time Use Inventory

We started by conducting exploratory and confirmatory factor analyses with 43 items (Mplus 8.8, MLR/MLM estimator, Geomin rotation). In interpreting the fit indices we relied on Hu and Bentler’s (1999) guidelines, using CFI values close to 0.95 or greater, RMSEA values close to 0.06 or below, and SRMR values close to 0.08 or below as evidence of reasonably good model fit. The fit indices for all the structural models are presented in Table 2.

**Table 2:** Fit indices for the structural models, Study 1.

Model	*χ*^2^ (df), *p*	CFI	TLI	RMSEA (90% CI)	SRMR
EFA, 4-factor model (43 items)	1235.97 (737), *p* < 0.001	0.819	0.775	0.063 [0.056, 0.069]	0.050
EFA, 4-factor model (22 items)	224.33 (149), *p* < 0.001	0.931	0.893	0.054 [0.039, 0.068]	0.037
CFA, 4-factor model (22 items)	306.22 (203), *p* < 0.001	0.907	0.895	0.054 [0.041, 0.066]	0.064
CFA, 4-factor model^*^ (22 items)	255.84 (201), *p* = 0.005	0.951	0.943	0.040 [0.023, 0.054]	0.060
CFA, hierarchical model^*^ (22 items)	275.80 (203), *p* < 0.001	0.935	0.926	0.046 [0.031, 0.059]	0.069

^*^Models including two additional error covariances for items 7 and 19, and 9 and 17.

In exploratory factor analyses, both parallel analysis and scree plot indicated four latent dimensions. The four dimensions were theoretically interpretable, reflecting self-congruent time use, balance of activities, sense of control over one’s time, and efficient time use. However, the fit of an initial EFA model with 43 items was poor. For each factor, we chose a subset of items with the most theoretically clear formulations and high factor loadings (*λ* > 0.50). The resulting set of 22 items exhibited an interpretable structure and a good fit to the data.

To find out the fit of the theoretically implied model without cross-loadings, we tested a conventional CFA measurement model which fit the data acceptably. We investigated the modification indices and found two strong outliers suggesting correlated uniquenesses for two pairs of items within the same scales. In one case, both items (Δ*χ*^2^ = 35.87, items 7 and 19) referred to “time for myself,” in the other case, both items (Δ*χ*^2^ = 14.09, items 9 and 17, placed adjacent in the original questionnaire) referred to the experienced meaninglessness of one’s daily activities. The resulting model with 2 additional covariances fit the data well. All the items had significant and substantial loadings on their respective factors (*λ* > 0.4).

**Figure 2:**
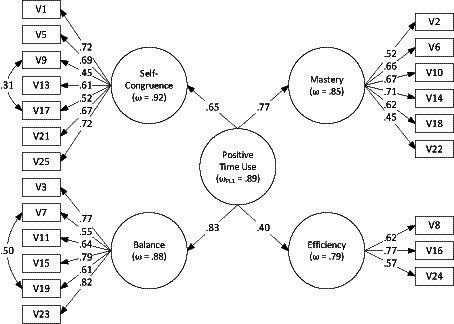
Parameters of the hierarchical CFA model, Study 1. Item labels correspond to Appendix; all the parameters shown are significant at *p* < 0.01.

To find out whether the 4 factors could be viewed as indicators of a single second-order dimension, we tested the fit of a higher-order model with a single second-order factor. Although the difference in the fit was significant, based on the scaled chi-square test (Δ*χ*^2^ = 19.97, *df* = 2, *p* < 0.001), the difference in the practical fit indices was quite small (ΔCFI = 0.016, ΔRMSEA = 0.006) and the practical fit indices were all within ranges indicating good model fit and supporting the possibility of viewing positive time use as a single second-order dimension. The parameters of the final CFA model are presented in Figure 2.

The descriptive statistics and reliability coefficients for the Positive Time Use Inventory scales are presented in Table 1. The findings indicate a good structural validity of the PTUI as a multifaceted measure with four subscales: Self-Congruence of Activities, Balance of Activities, Sense of Control over Time, and Efficiency of Time Use. However, the reliability of the Efficiency of Time Use subscale was modest, due to a small number of items, and only acceptable for research purposes. Also, one of the covariances we found could reflect an item order effect.

#### Positive time use and well-being

The correlations of the PTUI scales with well-being indicators are presented in Table 1. The PTUI scales were weakly to moderately correlated with each other. The findings indicate that the respondents who report positive time use tend to experience higher levels of subjective well-being and sense of coherence. At the same time, the correlations between these scales do not exceed 0.70, suggesting that the variance captured by the four dimensions of positive time use does not strongly overlap with that of well-being measures.

To investigate the variance shared by the individual dimensions of positive time use with well-being scales and to obtain preliminary evidence of the discriminant validity of the PTUI scales, we performed a multiple regression analysis. The 4 PTUI scales were entered simultaneously as predictors of each well-being measure. The results are presented in Table 3.

**Table 3:** Results of multiple regression analyses.

	Dependent variable					
	SWLS	PA	NA	SWB	SOC	SWTU
Variance explained, R^2^	0.42***	0.27***	0.25***	0.56***	0.51***	0.36***
*β* Self-congruence	0.46***	0.42***	−0.19*	0.51***	0.50***	0.14
*β* Balance	0.15*	0.09	−0.08	0.15*	0.11	0.23**
*β* Mastery	0.16**	0.00	−0.33***	0.22**	0.19**	0.23**
*β* Efficiency	0.06	0.12	−0.05	0.09	0.13*	0.24***

SWLS, Satisfaction with Life Scale; PA, Positive Affect; NA, Negative Affect; SWB, Subjective Well-Being (SWLS + PA – NA); SOC, Sense of Coherence; SWTU, Satisfaction with Time Use. ***p < 0.001, **p < 0.01, *p < 0.05.

Satisfaction with life was mainly associated with self-congruent time use, with a minor contribution of balance and control. Positive affect was only predicted by self-congruent time use. However, lack of sense of control over one’s time turned out to be the strongest predictor of negative affect. Sense of coherence was predicted by self-congruence, sense of control, and efficiency of time use. Finally, three out of four scales were significant predictors of the satisfaction with time use item, with the remaining self-congruence scale a marginally significant predictor (*p* = 0.062).

We found that self-congruence of activities, balance of activities, sense of control over one’s time, and perceived efficiency of time use show weak to moderate intercorrelations, suggesting discriminant validity. At the same time, all four subscales are significantly associated with subjective well-being and sense of coherence, indicating their convergent validity as well-being indicators. In multiple regression, we found three out of four PTUI scales to be significant predictors of self-reported satisfaction with time use (with the remaining scale being marginally significant), which indicates their validity as indicators of a multifaceted construct of time use satisfaction.

#### Limitations

The findings of Study 1 are in line with the expectations based on the theoretical model. However, the limitations of the study include modest sample size, which reduces the precision of parameter estimates (Kyriazos, 2018) and precludes from testing a more rigorous bifactor model (Bader et al., 2022), absence of a cross-validation sample for post-hoc model modifications, and, finally, the use of a student sample. Given these limitations, Study 1 can only be considered as a pilot study. In order to obtain more rigorous evidence in favor of the structure, as well as to test substantive hypotheses about the nomological network of positive time use, we conducted a second study presented below.

## Study 2: exploring the links between time perspective, time management, and time use

### Aim

Study 2 had multiple aims: (1) to improve the psychometric properties of PTUI by supplementing the number of items in the Efficiency subscale, (2) to cross-validate the structure of the measure in a larger and more representative sample, (3) to test the proposed theoretical model and to explore the nomological network of positive time use by investigating its associations with time perspective and time management.

### Method

#### Participants and procedure

The sample (*N* = 357) included 219 (61.3%) female and 138 (38.7%) male individuals, aged 17 to 71 (*M* = 40.19, *SD* = 11.07). The participants were employed or self-employed professionals who described their role in the company as top managers (33.2%), mid-level managers (37.1%) or team members (29.8%). In terms of education, most participants had completed a postgraduate degree (42.6%), an undergraduate degree (30.2%), or a professional diploma (12.6%), a minority had GCSE/A-levels (7.6%) or doctoral degrees (6.7%). The participants completed an anonymous online questionnaire on time use and well-being and received brief feedback on their scores. The questionnaire was designed to exclude missing responses.

#### Instruments

##### Positive Time Use Inventory

To improve the reliability of the Efficiency of Time Use scale, we supplemented it with three additional items (4, 12, and 20) derived from existing measures and tapping into inefficient use of time: “I find that during the day I am often not sure what to do next,” “I take a long time to ‘get going’,” and “I tend to change rather aimlessly from one activity to another during the day.” To reduce the possible response bias, we also slightly modified the instructions, asking participants to rate the extent to which each item is true about them using a 5-point Likert response scale from 1 (Very untrue) to 5 (Very true). The resulting version of the questionnaire is given in the Appendix.

##### Zimbardo Time Perspective Inventory (ZTPI)

The ZTPI (Zimbardo and Boyd, 1999) is a 56-item measure tapping into five dimensions of individual time perspective: Future (sample item: “I complete projects on time by making steady progress”), Present-Hedonistic (“I take risks to put excitement in my life”), Present-Fatalistic (“Since whatever will be will be, it does not really matter what I do”), Past-Positive (“I get nostalgic about my childhood”), and Past-Negative (“I think about the bad things that have happened to me in the past”). The items are rated on a 5-point Likert scale from 1 (Very untrue) to 5 (Very true).

In line with the theoretical arguments and empirical evidence showing that a balanced time perspective profile is a more important predictor of well-being than score on any single ZTPI dimension (Boniwell and Zimbardo, 2004; Boniwell et al., 2010), we also calculated the Deviation from Balanced Time Perspective (DBTP) index (Zhang et al., 2013; Stolarski et al., 2015b). This index reflects a Euclidean distance of an individual profile from a theoretically defined “optimal” one, characterized by a combination of high scores on the Future, Present-Hedonistic, and Past-Positive scales with low Present-Fatalistic and Past-Negative scores. There are numerous studies supporting its validity (Stolarski et al., 2020).

##### Time management behaviors

To further investigate the nomological network of positive time use, we chose four indicators of time management behaviors. We used three subscales from the Time Management Behavior Scale (TMBS) (Macan et al., 1990): Setting Goals and Priorities (sample item: “I review my goals to determine if they need revising”), Mechanics – Scheduling, Planning (“I write notes to remind myself of what I need to do”), and Preference for Organization (“At the end of the workday I leave a clear, well-organized workspace”). We also included a subscale from the Time Structure Questionnaire (TSQ) (Bond and Feather, 1988), Structured Routine (sample item: “My main activities during the day fit together in a structured way”). These items were rated using the same 5-point response scale going from 1 (Very untrue) to 5 (Very true).

We did not include the other TMBS and TSQ subscales, which tend to reflect either outcomes of time management behaviors and thus overlap in terms of item content with the positive time use construct (e.g., Perceived Control of Time from the TMBS, Sense of Purpose, Effective Organization from the TSQ, Present Orientation) or reflect the motivational processes underlying time management behaviors and thus overlap with the dimensions of the ZTPI (e.g., Present Orientation and Persistence from the TSQ).

Finally, to measure subjective well-being, we used the same versions of *Satisfaction with Life Scale* (Diener et al., 1985) and *Positive and Negative Affect Schedule* (Watson et al., 1988) as in Study 1.

The descriptive statistics and reliability coefficients for all the instruments used in this study are given in Table 4.

**Table 4:** Fit indices for the structural models, Study 2.

Model	*χ*^2^(df), *p* < 0.001	CFI	TLI	RMSEA [90% CI]	SRMR	AIC	BIC
ICM-CFA							
(1) 4 factors	496.00 (269)	0.920	0.911	0.049 [0.042, 0.055]	0.055	23115.05	23429.15
(2) Higher-order	558.42 (271)	0.898	0.888	0.055 [0.048, 0.061]	0.067	23181.72	23488.06
(3) Bifactor	492.00 (250)	0.915	0.897	0.052 [0.045, 0.059]	0.062	23145.93	23533.71
ICM-CFA modified*							
(4) 4 factors	423.36 (268)	0.945	0.939	0.040 [0.033, 0.047]	0.051	23033.23	23351.20
(5) Hierarchical	480.59 (270)	0.926	0.917	0.047 [0.040, 0.053]	0.064	23094.19	23404.41
(6) Bifactor	416.43 (249)	0.941	0.929	0.043 [0.036, 0.051]	0.057	23061.47	23453.12
ESEM							
(7) 4 factors	324.92 (206)	0.957	0.937	0.040 [0.032, 0.048]	0.030	23012.40	23570.80
(8) Hierarchical	314.00 (208)	0.962	0.945	0.038 [0.029, 0.046]	0.030	23008.75	23559.39
(9) Bifactor	251.77 (185)	0.976	0.961	0.032 [0.021, 0.041]	0.025	22976.00	23615.83

^*^Models including an additional covariance of items 7 and 19.

### Results and discussion

#### Structure of the Positive Time Use Inventory

To cross-validate the structure in the new sample and to investigate it more rigorously, taking advantage of the larger sample size, we performed a series of analyses in Mplus 8.8 using the new 25-item set with three additional items.

We tested three types models: a correlated-factor model with four factors, a more restricted higher-order model with a single second-order factor, and, finally, a bifactor model with a general positive time use factor and four specific factors reflecting the variance specific to items belonging to each subscale (Rodriguez et al., 2016). These models have different assumptions and advantages, notably, the higher-order model is the most parsimonious (as it assumes that the relationships of the general second-order factor with the indicators are fully mediated by the first-order factor) and can be seen as nested within the bifactor model, which provides a more detailed representation of the relationships between the variables, but can easily be overfit to the data, even if the model is misspecified (Markon, 2019).

Furthermore, each of these three models was tested, firstly, in its theoretically expected version, secondly, in a version with one additional covariance (replicated from Study 1), and, thirdly, following the Editor’s suggestion, as an ESEM model (Van Zyl and Ten Klooster, 2022) allowing all possible cross-loadings. The ESEM models allow to overcome the limitation of imperfect indicators, but, at the same time, are less constrained and easy to overfit, so that the choice between them and the more parsimonious models should not be based on the fit indices alone (Morin et al., 2020). The fit indices for all models are given in Table 4.

Using conventional criteria applicable to ICM-CFA models (Hu and Bentler, 1999) and based on the combination of fit indices (Brown, 2015), the theoretical ICM-CFA model (1) with four correlated factors showed an acceptable fit to the data. The standardized factor loadings ranged from 0.47 to 0.83 across the items (mean *λ* was 0.64 and ranged from 0.61 to 0.69 across the four scales), and the factor correlations ranged from 0.27 to 0.69.

Based on modification indices, we identified one correlated uniqueness of items 7 and 19 (Δ*χ*^2^ = 68.67). Both of these items refer to “time for myself” and belong to the Balance scale. The second correlated uniqueness found in Study 1 was not significant. All the remaining modification indices were comparatively weak with *χ*^2^ values of 12 or smaller, corresponding to expected STDYX parameter values of 0.28 or smaller for cross-loadings and error covariances. The resulting correlated-factor ICM-CFA model (4) with one error covariance fit the data quite well, based on all four practical fit indices (CFI and TLI close to 0.95, RMSEA and SRMR below 0.06). The standardized factor loadings ranged from 0.41 to 0.83 across the items (mean *λ* was again 0.64 and ranged from 0.61 to 0.69 across the four scales), the factor correlations ranged from 0.29 to 0.68.

**Figure 3:**
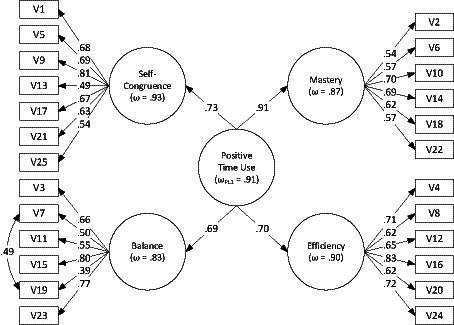
Parameters of the hierarchical CFA model, Study 2. Item labels correspond to Appendix; all the parameters shown are significant at *p* < 0.001.

The parameters of both correlated-factor models (1) and (4) are given in Supplementary Table S1. The parameters differed only marginally and the pattern of latent factor correlations (all going in one direction, statistically significant, and all but one comparable in magnitude) was consistent with the single higher-order dimension hypothesis. We followed by testing the more constrained model with a single second-order factor. Predictably, the model (2) only fit the data marginally, but the fit indices of the model (5) with an additional covariance were within ranges indicating good fit. Although the introduction of the single second-order dimension resulted in a significant decrease in model fit according to the scaled chi-square test (Δ*χ*^2^ = 75.84, *df* = 2, *p* < 0.001), the difference in practical fit indices was relatively small (ΔCFI = 0.019, ΔRMSEA = 0.007). The parameters of the resulting hierarchical model are shown in Figure 3. The loadings of all four scales on the second-order positive time use factor were all significant and pronounced (*λ* > 0.69), in line with a single construct hypothesis.

The four scales had good measurement reliability: the McDonald’s ω based on the hierarchical model ranged from 0.83 to 0.93 (see Figure 3) and Cronbach’s α for the observed sum scores ranged from 0.80 to 0.83 (see Table 5) for the subscales. The reliability of the total score was also high, with both α and ω = 0.91.

**Table 5:** Pearson correlations between Study 2 variables (*N* = 357).

	1	2	3	4	5	6	7	8	9	10	11	12	13	14	15	16	17	18
1. Self-congruence																		
2. Balance	0.46																	
3. Mastery	0.49	0.56																
4. Efficiency	0.52	0.20	0.55															
5. Positive time use index	0.80	0.70	0.84	0.75														
6. Past-Negative	−0.56	−0.39	−0.49	−0.37	−0.58													
7. Present-Hedonistic	0.10	0.18	0.05	−0.09	0.07	0.02												
8. Future	0.33	0.00	0.16	0.51	0.34	−0.10	−0.30											
9. Past-Positive	0.13	0.09	0.07	0.07	0.12	−0.11	0.03	0.09										
10. Present-Fatalistic	−0.39	−0.20	−0.31	−0.30	−0.39	0.41	0.30	−0.36	−0.09									
11. DBTP	−0.47	−0.29	−0.33	−0.34	−0.46	0.58	−0.05	−0.33	−0.70	0.55								
12. Mechanics	0.38	0.14	0.13	0.38	0.34	−0.09	−0.05	0.61	0.08	−0.25	−0.29							
13. Setting Goals	0.39	0.21	0.26	0.51	0.45	−0.12	−0.07	0.66	0.15	−0.26	−0.35	0.64						
14. Pref. for Organization	0.32	0.15	0.34	0.49	0.43	−0.27	−0.27	0.54	0.15	−0.37	−0.35	0.47	0.48					
15. Structured Routine	0.29	0.18	0.20	0.43	0.36	−0.06	−0.08	0.54	0.09	−0.16	−0.21	0.52	0.60	0.44				
16. Positive Affect	0.60	0.36	0.38	0.35	0.55	−0.39	0.27	0.14	0.11	−0.22	−0.34	0.28	0.30	0.13	0.18			
17. Negative Affect	−0.31	−0.29	−0.46	−0.25	−0.42	0.46	0.10	−0.11	−0.08	0.28	0.32	0.01	−0.04	−0.22	0.01	−0.20		
18. Satisfaction with Life	0.56	0.40	0.36	0.31	0.52	−0.57	0.11	0.17	0.19	−0.19	−0.41	0.21	0.24	0.21	0.17	0.38	−0.24	
M	3.88	3.47	3.14	3.36	3.48	2.52	3.35	3.57	3.35	2.32	2.10	3.31	3.44	3.41	3.10	3.71	1.98	4.95
SD	0.65	0.73	0.76	0.84	0.57	0.71	0.51	0.50	0.64	0.63	0.68	0.68	0.63	0.70	0.69	0.64	0.66	1.22
α	0.83	0.80	0.80	0.84	0.91	0.84	0.79	0.74	0.77	0.75	--	0.78	0.81	0.77	0.63	0.84	0.83	0.87

Correlations with magnitude | r | ≥ 0.11 are significant at *p* < 0.05, | r | ≥ 0.14 *p* < 0.01, | r | ≥ 0.18 *p* < 0.001.

To further investigate the structure of the scale, we also tested the bifactor model and calculated the corresponding set of indices (Rodriguez et al., 2016). Predictably, the bifactor models (3) and (6) also fit the data well, although all the practical fit indices and the information criteria tended to favor the more parsimonious correlated-factor versions. As in the case with correlated-factor models, the parameter estimates and the values of bifactor model indices only differed very slightly between the models with and without the additional covariance; for brevity we only present the parameters of the “classical” bifactor model (3) in Table 6.

**Table 6:** Factor loadings and reliability coefficients based on the bifactor ESEM model (9) and the bifactor ICM-CFA model (3), Study 2.

Item	Positive time use index	Self-congruence	Balance	Mastery	Efficiency
V1	0.42* (0.50*)	0.42* (0.46*)	−0.04	−0.03	0.09
V5	0.40* (0.49*)	0.47* (0.51*)	0.06	−0.09	−0.03
V9	0.52* (0.55*)	0.71* (0.65*)	0.05	0.10	0.06
V13	0.40* (0.49*)	0.18* (0.16*)	0.05	−0.08	0.01
V17	0.50* (0.53*)	0.45* (0.40*)	0.04	0.03	0.20*
V21	0.42* (0.61*)	0.25* (0.23*)	0.03	0.01	0.00
V25	0.47* (0.46*)	0.32* (0.27*)	0.01	0.01	−0.05
V3	0.55* (0.53*)	0.05	0.38* (0.34*)	0.03	−0.16
V7	0.19 (0.32*)	0.19*	0.82* (0.66*)	0.42*	0.03
V11	0.49* (0.50*)	−0.01	0.19* (0.18*)	−0.03	−0.10
V15	0.68* (0.63*)	0.02	0.37* (0.35*)	−0.03	−0.27*
V19	0.15 (0.17*)	0.02	0.67* (0.71*)	0.28*	−0.07
V23	0.64* (0.60*)	−0.05	0.39* (0.34*)	−0.11	−0.20*
V2	0.44* (0.44*)	−0.06	0.19	0.24* (0.26*)	0.16
V6	0.48* (0.36*)	−0.08	0.01	0.64* (0.61*)	−0.12
V10	0.64* (0.58*)	0.09	0.05	0.41* (0.33*)	0.17
V14	0.65* (0.56*)	−0.02	0.08	0.47* (0.43*)	0.04
V18	0.51* (0.57*)	−0.05	0.12	0.15 (0.19*)	0.17
V22	0.54* (0.44*)	0.03	0.06	0.43* (0.38*)	0.10
V4	0.51* (0.51*)	0.11	−0.07	0.18*	0.47* (0.48*)
V8	0.49* (0.35*)	−0.14	−0.15	0.04	0.54* (0.53*)
V12	0.54* (0.41*)	−0.01	−0.18	0.05	0.47* (0.51*)
V16	0.69* (0.54*)	0.08	−0.21*	−0.02	0.70* (0.64*)
V20	0.43* (0.39*)	0.16	−0.15*	0.27*	0.48* (0.47*)
V24	0.58* (0.49*)	0.10	−0.09	0.02	0.55* (0.52*)
ω/ω_S_	0.93 (0.93)	0.84 (0.84)	0.84 (0.81)	0.83 (0.79)	0.85 (0.85)
ω_H_/ω_HS_	0.78 (0.77)	0.37 (0.29)	0.40 (0.38)	0.31 (0.28)	0.40 (0.49)

*p < 0.01; the theoretically expected factor loadings are marked with bold, the values for the ICM-CFA bifactor model (3) are given in parentheses. Item codes correspond to the Appendix. All the reverse-scored items (2, 4, 6, 7, 9, 10, 12, 14, 16, 17, 20, 22, 24) were inverted. The bifactor structure coefficients were calculated using the R BifactorIndicesCalculator package.

In this model (3), all the factor loadings of items on the general factor and on their respective specific factors were statistically significant. The Explained Common Variance (ECV) index was 0.54, indicating that only about half of the non-error variance of items is common to all the subscales, and suggesting that treating the positive time use construct as multidimensional might reveal some effects peculiar to its individual dimensions. The omega coefficient was 0.93 for the general factor and ranged from 0.79 to 0.85 for the four subscales, indicating good reliability based on joint contribution of the general factor and the subscale-specific variance. The hierarchical omega coefficients for the subscales ranged from 0.28 to 0.49, indicating a substantial proportion of reliable subscale-specific variance remaining after partitioning out the contribution of the general factor. This proportion was the highest for the Efficiency subscale. These analyses based on the bifactor model provide evidence in favor of discriminant validity of the subscales.

Finally, we tested the ESEM versions of the three models. Even though the correlated-factor ESEM model (7) had extremely good fit indices, the structure obtained after target rotation did not approximate simple structure (see Supplementary Table S1): there were many substantial cross-loadings and the correlations between factors had different signs. Predictably, the hierarchical ESEM model (8) tested using the procedure proposed by Morin and Asparouhov (2018), despite showing good fit indices, failed to converge on a proper solution indicating that this correlation structure could not be represented by a single higher-order dimension. Nevertheless, the bifactor ESEM model (9) has shown good convergence and superior values of practical fit indices, despite the higher Bayesian Information Criterion (BIC) scores, compared to the ICM-CFA models (3) and (6).

A substantive comparison of the parameters of models (3) and (9) (see Table 6) shows that their differences are quite small: there are only a few cross-loadings unaccounted for by the bifactor model. Apart from the covariance of items 7 and 19 being assigned to the Mastery factor, the other cross-loadings were weak. The loadings of items on the general and group factors, as well as the values of indices based on the bifactor model were comparable to those obtained using the more parsimonious model (3). The only minor differences concerned the estimates of subscale-specific variance for the Self-Congruence and Efficiency factors. Thus, the results of bifactor ESEM have corroborated the results of ICM-CFA modeling, and supported the expected structure.

#### Does positive time use explain the effects of time perspective on well-being?

First, we explored the correlational links between positive time use, time perspective, and well-being measures (see Table 5). Past-Negative and Present-Fatalistic perspectives were negatively associated to all four PTUI dimensions. Their associations with self-congruent time use were particularly strong, suggesting that these perspectives are associated with inability to choose satisfying activities. Future time perspective was positively related to the efficiency of time use, self-congruence of activities, and a sense of mastery over one’s time. The associations of Present-Hedonistic and Past-Positive perspectives with positive time use were only marginal. The pattern of associations between PTUI and SWB indicators was similar to that found in Study 1.

For more detail, the correlations of the factor scores derived from the bifactor models separating the general effects of positive time use and the specific effects of residualized subscale factors are given in Supplementary Table S3. Below we will focus only on the combined variance of the positive time use dimensions, aiming to validate the theoretical model.

To test the substantive hypothesis 2 that positive time use mediates the associations of time perspective and well-being, we tested a path model in Mplus, where the ZTPI scores predicted subjective well-being and their effects were partially mediated by positive time use. To simplify the model, we used an aggregate subjective well-being score and the total PTUI score. To evaluate the model, we used Maximum Likelihood estimator with 1,000 bootstrap samples to compute the 95% confidence intervals for the indirect effects. To assess the effect size of the mediation, we used the relative indirect effect index P_M_, which reflects the ratio of the indirect effect magnitude to that of the total effect (Preacher and Kelley, 2011).

**Figure 4:**
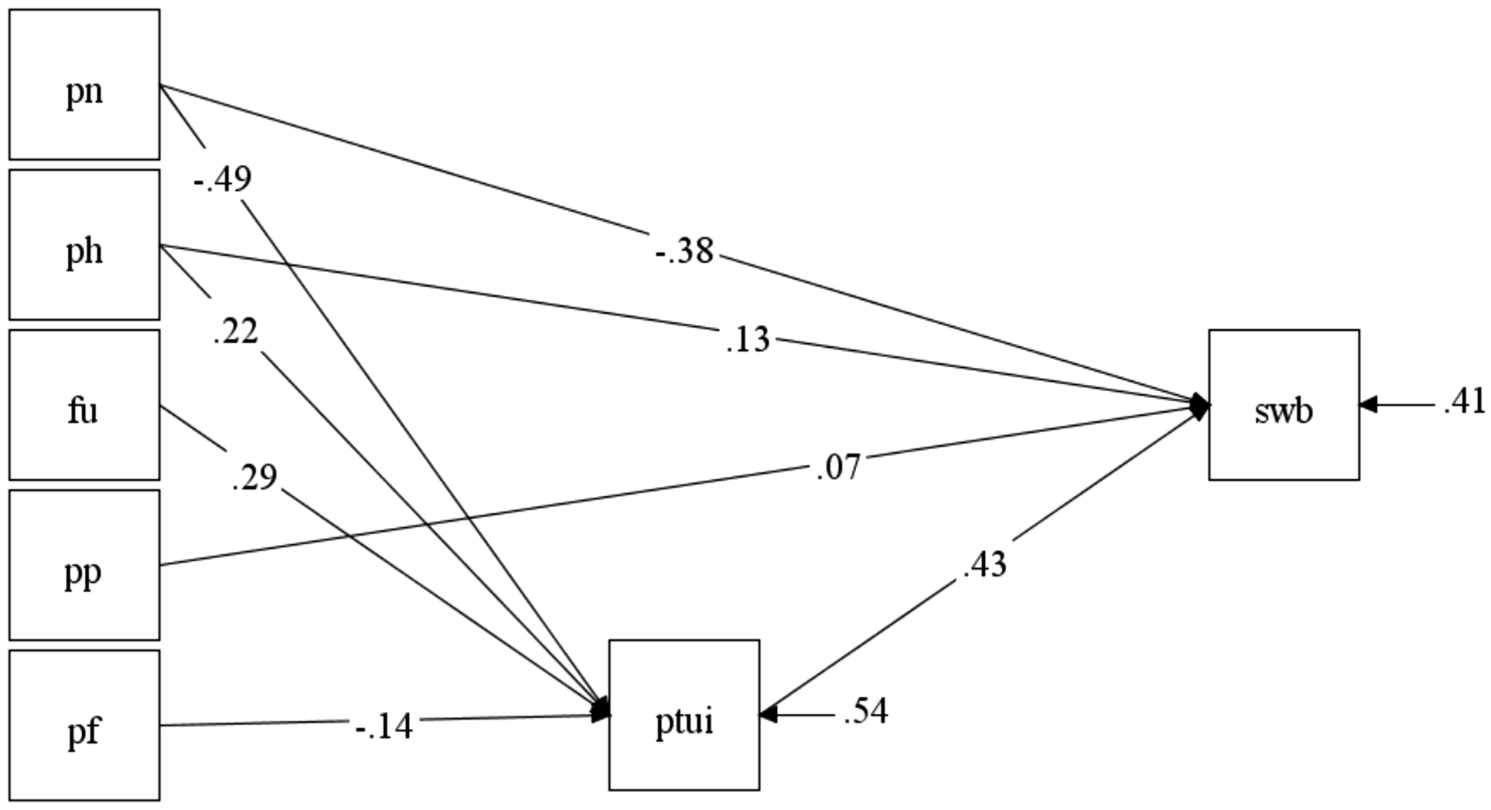
Positive time use as a mediator of the associations between time perspective and subjective well-being. Saturated model; parameters significant at *p* < 0.05 are shown. PN, Past-Negative; PF, Present-Fatalistic; FU, Future; PH, Present-Hedonistic; PP, Past-Positive; PTUI, Positive Time Use; SWB, Subjective Well-Being.

The resulting model is presented in Figure 4. Positive time use emerged as a full mediator of the effects of the Future (*β*_ind_ = 0.13 [0.08; 0.18], *p* < 0.001, P_M_ = 0.77) and Present-Fatalistic (*β*_ind_ = −0.06 [−0.11; −0.02], *p* < 0.01, P_M_ = 0.74) time perspectives. The absence of significant direct effects suggests that the individual differences in well-being associated with future orientation and fatalistic orientation toward the present fatalistic are fully explained by the variance in positive time use. We only found partial mediation for the effects of past-negative (*β*_ind_ = −0.22 [−0.27; −0.15], *p* < 0.001; P_M_ = 0.36) and present-hedonistic (*β*_ind_ = 0.10 [0.06; 0.14], *p* < 0.001; P_M_ = 0.43). Finally, past-positive had only a positive direct effect on SWB.

When the DBTP index was used as a predictor instead of the individual ZTPI scales, we found a partial mediation with indirect effect (*β*_ind_ = −0.28 [−0.33; −0.22], *p* < 0.001; P_M_ = 0.56) being somewhat stronger than the direct effect (*β* = −0.22 [−0.31; −0.14], *p* < 0.001), suggesting that the shared variance of balanced time perspective and well-being is largely, but not completely, explained by the variance in positive time use.

#### Does time management explain the effects of time perspective on positive time use?

Based on the above findings, we sought to test the hypothesis 3 stating that time management explains the effects of time perspective on positive time use and that the latter explains the effects of time management on well-being. First, we tested whether the four scales tapping into time management behaviors could be treated as a single dimension. A confirmatory factor analytic model with a single factor fit the data perfectly (*χ*^2^(2) = 2.07, *p* = 0.25, CFI > 0.999, RMSEA = 0.010 [0.000; 0.106], SRMR = 0.010), with standardized factor loadings of 0.60 (Preference for Organization), 0.71 (Structured Routine), 0.76 (Mechanics – Scheduling, Planning), and 0.83 (Setting Goals and Priorities).

Next, we tested a model where the 5 ZTPI scales were entered as predictors of the latent factor of time management behaviors. The model fit the data acceptably (*χ*^2^(17) = 55.92, *p* < 0.001, CFI = 0.946, RMSEA = 0.080 [0.057; 0.104], SRMR = 0.057). The 5 time perspective dimensions explained together 68% of the variance in time management. However, Future time perspective emerged as the only important predictor (*β* = 0.81, *p* < 0.001) explaining 66% of the variance in time management behaviors. The only other significant predictor was Present-Hedonistic (*β* = 0.13, *p* = 0.008), but its contribution to variance explained was extremely small, and we opted to model only the effects of the Future time perspective.

Finally, to test the third substantive hypothesis, we tested a path model with serial mediation, where the Future time perspective predicted time management, which fully mediated its effects on positive time use, which, in turn, fully mediated the effects of time management on subjective well-being. To simplify the model, the four time management variables were standardized and combined into a single index; the same approach was used for subjective well-being based on satisfaction with life, positive affect, and negative affect scales.

**Figure 5:**
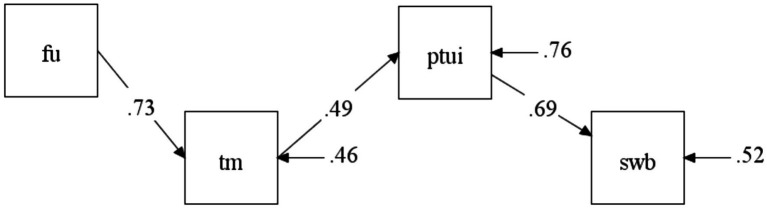
Time management and positive time use as mediators of the effects of future time perspective on well-being. Model fit: *χ*^2^(3) = 3.32, *p* = 0.35, CFI = 0.999, RMSEA = 0.001 [0.010; 0.093], SRMR = 0.024. FU, Future time perspective scale (ZTPI); TM, Time management index; PTUI, Positive Time Use Inventory total score; SWB, Subjective well-being index.

The parameters of the full mediation model tested are shown in Figure 5. The model fit the data perfectly, according to the chi-square test of exact fit. The indirect effect of future time perspective on positive time use mediated by time management was significant (*β*_ind_ = 0.36 [0.29; 0.43], *p* < 0.001). The indirect effect of future time perspective on subjective well-being mediated by time management behaviors and positive time use was also significant (*β*_ind_ = 0.25 [0.20; 0.31], *p* < 0.001). The findings suggest that the higher levels of well-being found in individuals with future time perspective can be explained by their time management behaviors and better satisfaction with their use of time.

## General discussion

The model of positive time use is based on phenomenological data obtained from semi-structured interviews by the second author who sought to describe and understand the phenomenon of satisfaction with the use of time. The findings of the two quantitative studies described above demonstrate the structural, convergent, and discriminant validity of the new measure, Positive Time Use Inventory, which operationalizes the theoretical model based on qualitative findings. In structural and convergent validity analyses the four dimensions of positive time use emerge as interrelated, yet distinct: even though they all describe different aspects of satisfying time use, they have substantial proportions of non-shared variance and show different patterns of association with well-being outcomes.

In structural analyses, we relied on hierarchical and bifactor models. We saw good fit both of hierarchical models that provide a more parsimonious representation of the structure and bifactor models, which allow for a more detailed partitioning of the variance, are based on an assumption of a single underlying construct, but also require larger sample sizes (Markon, 2019; Bader et al., 2022). The results of bifactor ICM-CFA and bifactor ESEM showed very good convergence, supporting the potential discriminant validity of the subscales.

Theoretically, the construct of positive time use fills the gap between time perspective, on one hand, and well-being, on the other hand, by elucidating some of the processes and mechanisms behind their associations. Although positive time use only partially mediates the effects of balanced time perspective with well-being, it fully explains the effects of the Future and Present-Fatalistic dimensions of time perspective and largely explains the effects of the Present-Hedonistic dimension. These findings suggest that satisfaction with time use and subjective well-being can be facilitated by adopting a conscious, mindful, and responsible approach to the time we have at our disposal. Future studies could explore whether the coaching and therapy interventions aiming to achieve a balanced time perspective have causal effects on positive time use.

The concept of positive time use has also allowed us to explore and explain the links of time management with time perspective, on the one hand, and with well-being, on the other hand. We found that time management behaviors are chiefly predicted by future time perspective, in line with previous studies (Bajec, 2019). The findings supported our theoretical model suggesting that time management behaviors fully mediate the effects of future perspective on positive time use, which, in turn, explains their associations with well-being. Unfortunately, cross-sectional design precludes us from making any causal inferences at this stage. However, the working model proposed above paves the way for more rigorous longitudinal studies that could find out whether these links are indeed causal and establish their direction.

The results from the student sample (Study 1) and the working adult sample (Study 2) indicate that satisfying time use and efficient time use do indeed come in combination. Nevertheless, some individual differences in the relative importance of the facets of positive time use can be expected. Individuals focused on achievement might use their time efficiently in pursuit of important goals at the expense of balance between life spheres: past findings indicate that work-life balance is more important for the well-being of working adults than for those who are not employed (Dittrich and Mey, 2015). The results of additional exploratory analyses using person-oriented approach (see Supplementary Table S4) suggest that individuals with a strong future orientation and those with a more balanced time perspective emphasizing Present-Hedonistic have different profiles, yet comparable levels of positive time use, as well as comparable levels of general well-being. However, this might only be true for the achievement-oriented business employees comprising our Study 2 sample. Does sacrificing one’s life balance for the sake of one’s future goals come at a price and, if yes, does this price become evident with time? Longitudinal studies using larger and more diverse samples are needed to further explore the individual differences in positive time use patterns.

The findings also suggest that time management behaviors are mainly supported by future time perspective and might mainly be beneficial for positive time use and overall well-being in future-oriented individuals. This is in line with existing evidence showing that the effects of time management interventions are generally modest and may differ vastly depending on the individual and group contexts (Aeon and Aguinis, 2017). More research is needed to find out whether the efficient time use associated with a predominance of future orientation and active time management strategies does indeed translate into higher achievement and whether it is sustainable in a long term.

Naturally, the present studies are limited by modest sample sizes and cross-sectional designs. Given the known cultural differences with respect to the subjective notions of time (Meyer, 2016), the pace of life (Levine and Norenzayan, 1999), and time perspective (Sircova et al., 2015), it is quite possible that some of the effects that we discovered may be peculiar to the British culture and/or to professionally active adults. After all, the very concept of positive time use is operationalized based on the phenomenological data from only one country, Great Britain. Future qualitative studies could tap into the subjective notions of time spent well across different cultural contexts.

Nevertheless, the findings provide sound evidence concerning the structural, convergent, and discriminant validity of the Positive Time Use Inventory in two different UK samples, as well as some information about the nomological network of the new construct. The PTUI emerges as a promising new tool that can be used as part of time perspective coaching process with individuals and organizations (Boniwell et al., 2014; Boniwell and Osin, 2015) in order to diagnose the individual problems and to evaluate the effects of time coaching and time management interventions.

The results concerning the effects of time perspective and time management on positive time use suggest that efficient time use can be facilitated by strengthening one’s future time perspective and adopting time management strategies. However, achieving a satisfying time use might rather require a productive relationship with the present – the capacity to enjoy the present moment, to seize the emerging opportunities, and to overcome a passive, fatalistic attitude to life. Thus, the balance facet of positive time use can be facilitated by adopting a more balanced time perspective. Intervention studies could test whether these different approaches to positive time use can complement each other in the coaching process and whether ‘smart’ time management coaching interventions taking into account the individual differences in time perspective and positive time use could be more effective than the ‘one-size-fits-all’ time management recipes.

## Data availability statement

The raw data supporting the conclusions of this article will be made available by the authors, without undue reservation.

## Ethics statement

The studies involving humans were approved by the Open University Human Participants and Materials Ethical Committee. The studies were conducted in accordance with the local legislation and institutional requirements. The participants provided their written or online informed consent to participate in this study.

## Author contributions

EO and IB contributed to the conception and design of the study and performed the data collection. EO performed the statistical analyses and wrote the first draft of the manuscript. All authors contributed to manuscript revision, read, and approved the submitted version.

## Appendix

**Positive Time Use Inventory**

Please read each item and, as honestly as you can, answer the question: “How characteristic or true is this of you?” Please give your answer by choosing a number:

**Table:**

Very untrue	Mostly untrue	Neutral	Mostly true	Very true
1	2	3	4	5
				
Looking at a typical day in my life, I think that most things I do have some purpose.				1 2 3 4 5
I often do not have enough time to accomplish my tasks.				1 2 3 4 5
I am comfortable with the work-life boundaries in my life.				1 2 3 4 5
I find that during the day I am often not sure what to do next.				1 2 3 4 5
I like what I do.				1 2 3 4 5
I am often anxious about time.				1 2 3 4 5
I never have time for myself.				1 2 3 4 5
I rarely procrastinate.				1 2 3 4 5
I often feel that what I do is meaningless.				1 2 3 4 5
I feel time is slipping away through my fingers.				1 2 3 4 5
I have a balance between what I want to do and what I have to do.				1 2 3 4 5
I take a long time to ‘get going’.				1 2 3 4 5
I live my life in accordance with my goals.				1 2 3 4 5
I feel overwhelmed by my responsibilities.				1 2 3 4 5
I feel I have a balance of activities in my life.				1 2 3 4 5
I waste a lot of time.				1 2 3 4 5
My daily activities often seem trivial and unimportant to me.				1 2 3 4 5
I usually feel in control of my time.				1 2 3 4 5
I take some time for myself on a daily basis.				1 2 3 4 5
I tend to change rather aimlessly from one activity to another during the day.				1 2 3 4 5
I feel I am making progress.				1 2 3 4 5
I often feel guilty about not having done something.				1 2 3 4 5
I feel that different areas of my life are well-balanced.				1 2 3 4 5
Days often go by without me having done a thing.				1 2 3 4 5
If I should die today, I would feel that my life has been worthwhile.				1 2 3 4 5

Key (* indicates reverse-scored items, keyed 5 4 3 2 1): Self-Congruence: items 1, 5, 9*, 13, 17*, 21, 25; Balance: items 3, 7*, 11, 15, 19, 23; Efficiency: items 4*, 8, 12*, 16*, 20*, 24*; Mastery: items 2*, 6*, 10*, 14*, 18, 22*; Positive time use index: items 1–25 (after inverting all the reverse-scored items).
