# Supplementary material for: Positive time use: a missing link between time perspective, time management, and well-being
Source: Front Psychol. 2024 Feb 26;15:1087932. doi: 10.3389/fpsyg.2024.1087932 (PMC10927010; doi:10.3389/fpsyg.2024.1087932)
Supplement: Supplementary file 1 [file Data_Sheet_1.docx]

**Supporting Information**

**1. Structure of the PTUI: Correlated-factor models**

**Table SI.1**

*Factor loadings, correlations, and reliability coefficients based on the correlated-factor models (1) and (4) (Study 2)*

| Item | Self-congruence | Balance | Mastery | Efficiency |
| --- | --- | --- | --- | --- |
| V1 | .68 (.68) |  |  |  |
| V5 | .69 (.70) |  |  |  |
| V9 | .80 (.80) |  |  |  |
| V13 | .50 (.50) |  |  |  |
| V17 | .68 (.68) |  |  |  |
| V21 | .63 (.63) |  |  |  |
| V25 | .54 (.54) |  |  |  |
| V3 |  | .66 (.66) |  |  |
| V7 |  | .57 (.52) |  |  |
| V11 |  | .53 (.54) |  |  |
| V15 |  | .78 (.79) |  |  |
| V19 |  | .47 (.41) |  |  |
| V23 |  | .74 (.75) |  |  |
| V2 |  |  | .55 (.55) |  |
| V6 |  |  | .56 (.56) |  |
| V10 |  |  | .68 (.69) |  |
| V14 |  |  | .69 (.69) |  |
| V18 |  |  | .63 (.63) |  |
| V22 |  |  | .57 (.57) |  |
| V4 |  |  |  | .71 (.71) |
| V8 |  |  |  | .62 (.62) |
| V12 |  |  |  | .65 (.65) |
| V16 |  |  |  | .83 (.83) |
| V20 |  |  |  | .63 (.63) |
| V24 |  |  |  | .71 (.71) |
| ω | .84 (.84) | .80 (.75) | .78 (.78) | .85 (.85) |
| Factor correlations |  |  |  |  |
| Self-Congruence |  | .59 (.60) | .59 (.59) | .59 (.59) |
| Balance |  |  | .69 (.68) | .27 (.29) |
| Mastery |  |  |  | .68 (.68) |

*Note*: All the parameters are significant at p < .001. Item codes correspond to the Appendix. All the reverse-scored items (2, 4, 6, 7, 9, 10, 12, 14, 16, 17, 20, 22, 24) were inverted. The parameters for the model (4) with additional error covariance of items 7 and 19 (r = .48) are given in parentheses. Omega coefficients were calculated using the Jöreskog (1971) formulae cited in Brown (2015, formulae 8.1 and 8.2).

**2. Structure of the PTUI: Correlated-factor ESEM model**

**Table SI.2**

*Factor loadings and reliability coefficients based on the four-factor ESEM model (7) (Study 2)*

| Item | Self-congruence | Balance | Mastery | Efficiency |
| --- | --- | --- | --- | --- |
| V1 | **.78*** | .00 | -.29* | .19 |
| V5 | **.94*** | .17 | -.43* | -.08 |
| V9 | **.96*** | .19 | -.48* | .14 |
| V13 | **.48*** | .16 | .04 | .02 |
| V17 | **.66*** | .07 | -.16 | .28* |
| V21 | **.60*** | .21* | -.02 | .08 |
| V25 | **.57*** | .18* | -.09 | .02 |
| V3 | .24* | **.64*** | .38* | -.23* |
| V7 | -.03 | **.93*** | .28 | -.13 |
| V11 | .21 | **.43*** | .39* | -.13 |
| V15 | .33* | **.74*** | .47* | -.36* |
| V19 | -.16 | **.84*** | .33 | -.20 |
| V23 | .26 | **.70*** | .55* | -.34* |
| V2 | -.11 | .35* | **.56*** | .30* |
| V6 | -.16 | .44* | **.51*** | .26* |
| V10 | .08 | .28* | **.46*** | .43* |
| V14 | -.01 | .41* | **.57*** | .33* |
| V18 | .00 | .27* | **.60*** | .34* |
| V22 | -.01 | .30* | **.45*** | .35* |
| V4 | .14 | -.14 | .37* | **.72*** |
| V8 | -.09 | -.31* | .57* | **.69*** |
| V12 | .09 | -.27* | .42* | **.64*** |
| V16 | .21 | -.39* | .42* | **.83*** |
| V20 | .11 | -.20* | .23 | **.73*** |
| V24 | .20 | -.24* | .36* | **.67*** |
| ω | .71 | .70 | .78 | .72 |
| Factor correlations |  |  |  |  |
| Self-Congruence |  | -.12 | .67* | .01 |
| Balance |  |  | -.44* | .63* |
| Mastery |  |  |  | -.35* |

*Note*: * p < .01; the theoretically expected factor loadings are marked with bold. Item codes correspond to the Appendix. All the reverse-scored items (2, 4, 6, 7, 9, 10, 12, 14, 16, 17, 20, 22, 24) were inverted. N = 357, χ^2^(250) = 492.00, CFI = .915, RMSEA = .052, 90% CI [.045, .059], SRMR = .062. Omega coefficients were calculated using the Jöreskog (1971) formula cited in Brown (2015, formulae 8.1 and 8.2).

**3. Correlations of the general and specific**

**Table SI.3**

*Correlations of latent factor score estimates based on the bifactor model (6) and bifactor-ESEM model (9) with other variables*

| Observed scores | Bifactor model (6) | | | | | Bifactor ESEM model (9) | | | | |
| --- | --- | --- | --- | --- | --- | --- | --- | --- | --- | --- |
|  | FG | SC | BA | MA | EF | FG | SC | BA | MA | EF |
| 1. Positive Time Use Index | .98 | .25 | .22 | .20 | .23 | .92 | .26 | .21 | .29 | .27 |
| 2. Self-congruence | .77 | .73 | .12 | -.09 | .07 | .73 | .71 | .05 | -.02 | .07 |
| 3. Balance | .65 | .09 | .78 | .15 | -.28 | .66 | .06 | .74 | .15 | -.20 |
| 4. Mastery | .87 | -.09 | .08 | .55 | .04 | .76 | -.02 | .13 | .63 | .13 |
| 5. Efficiency | .72 | .06 | -.22 | .02 | .79 | .68 | .07 | -.19 | .13 | .75 |
| 6. Past-Negative | -.56 | -.25 | -.14 | -.21 | -.05 | -.56 | -.25 | -.03 | -.21 | .00 |
| 7. Present-Hedonistic | .07 | .09 | .23 | .04 | -.19 | .11 | .05 | .14 | -.02 | -.23 |
| 8. Future | .33 | .13 | -.19 | -.21 | .43 | .31 | .12 | -.15 | -.12 | .43 |
| 9. Past-Positive | .11 | .08 | .11 | .03 | -.02 | .15 | .04 | .01 | -.02 | -.06 |
| 10. Present-Fatalistic | -.41 | -.19 | .01 | .01 | -.05 | -.33 | -.24 | -.06 | -.10 | -.13 |
| 11. DBTP | -.45 | -.24 | -.14 | -.02 | -.06 | -.46 | -.22 | -.04 | -.04 | -.03 |
| 12. Mechanics | .33 | .23 | -.01 | -.24 | .27 | .33 | .21 | -.02 | -.18 | .27 |
| 13. Setting Goals | .45 | .06 | -.03 | -.16 | .35 | .43 | .06 | -.02 | -.07 | .36 |
| 14. Pref. for Organization | .44 | .00 | -.14 | -.01 | .32 | .35 | .06 | -.01 | .14 | .40 |
| 15. Structured Routine | .36 | .04 | -.02 | -.18 | .30 | .34 | .04 | .01 | -.09 | .34 |
| 16. Positive Affect | .55 | .36 | .15 | -.02 | .03 | .54 | .35 | .05 | .01 | .01 |
| 17. Negative Affect | -.40 | -.06 | -.05 | -.31 | .00 | -.35 | -.10 | -.10 | -.36 | -.03 |
| 18. Satisfaction with Life | .52 | .29 | .26 | -.05 | .00 | .57 | .23 | .08 | -.11 | -.06 |
| Factor score parameters |  |  |  |  |  |  |  |  |  |  |
| Determinacy | .91 | .81 | .76 | .81 | .91 | .94 | .86 | .88 | .81 | .85 |
| SE |  |  |  |  |  | .35 | .52 | .48 | .59 | .53 |
| Factor score correlations |  |  |  |  |  |  |  |  |  |  |
| Bifactor model (6) FG |  | .18 | .15 | .16 | .18 | .91 | .22 | .17 | .30 | .26 |
| Bifactor model (6) SC |  |  | .02 | -.24 | -.05 | .19 | .94 | -.07 | -.24 | -.12 |
| Bifactor model (6) BA |  |  |  | .04 | -.44 | .36 | -.14 | .64 | -.23 | -.56 |
| Bifactor model (6) MA |  |  |  |  | -.14 | .17 | -.24 | -.05 | .77 | -.23 |
| Bifactor model (6) EF |  |  |  |  |  | .21 | -.09 | -.42 | -.13 | .86 |
| Bifactor model (9) FG |  |  |  |  |  |  | .09 | .05 | .05 | .09 |
| Bifactor model (9) SC |  |  |  |  |  |  |  | .02 | -.03 | .00 |
| Bifactor model (9) BA |  |  |  |  |  |  |  |  | .15 | -.14 |
| Bifactor model (9) MA |  |  |  |  |  |  |  |  |  | .08 |

*Note*. Correlations with magnitude | r | ≥ .11 are significant at p < .05, | r | ≥ .14 p < .01, | r | ≥ .18 p < .001. FG – General Positive Time Use Factor, specific factors: SC – Self-Congruence, BA – Balance, MA – Mastery, EF – Efficiency.

The correlations of the factor scores derived from the bifactor models separating the general effects of positive time use and the specific effects of residualized subscale factors are given in Table SI.4. In terms of well-being, Self-Congruence and Balance dimensions reveal some common specific variance with positive affect and satisfaction with life, whereas the Mastery dimension is inversely related to negative affect. The subscale factors also have some specific common variance with individual time perspective dimensions, notably, Efficiency is related to the future time perspective, Balance to Present-Hedonistic, and Self-Congruence is inversely related to Past-Negative. Finally, the time management dimensions reveal some common variance specific to the Efficiency subscale. In most cases, however, the effects related to the general factor are stronger than those related to specific factors.

**SI.4. Time perspective profiles and positive time use**

Previous studies indicate that the effects of each particular time perspective dimension measured by the ZTPI are less important than the time perspective profile (Boniwell et al., 2010). Based on this, we performed an additional analysis using person-oriented approach to investigate how different profiles of time perspective predict positive time.

To establish the time perspective profiles, we performed hierarchical cluster analysis (Ward’s method, Squared Euclidean metric) using standardized scores on the 5 ZTPI scales. Based on the elbow plot, we chose a model with 5 clusters. Next, we used One-way ANOVA with Tukey HSD post-hoc test to interpret the differences between groups. The standardized scores on the dependent variables in each cluster are presented in Table SI.4.

**Table SI.4**

*Standardized mean scores for the time perspective clusters*

|  | Risk-taking | Negative | Diffuse | Future-oriented | Balanced | Effect size, η^2^ |
| --- | --- | --- | --- | --- | --- | --- |
|  | (N = 50) | (N = 114) | (N = 70) | (N = 66) | (N = 57) |  |
| Time Perspective: DBTP | 0.99_c_ | 0.08_b_ | 0.29_b_ | -0.49_a_ | -0.82_a_ | .31 |
| Past-Negative | 0.85_b_ | 0.54_b_ | -0.62_a_ | -0.41_a_ | -0.59_a_ | .35 |
| Present-Hedonistic | 1.07_c_ | -0.31_b_ | -0.20_b_ | -0.89_a_ | 0.95_c_ | .49 |
| Future | -1.02_a_ | 0.19_c_ | -0.29_b_ | 0.99_d_ | -0.29_b_ | .37 |
| Past-Positive | -0.08_b_ | 0.15_bc_ | -0.93_a_ | 0.35_c_ | 0.51_c_ | .25 |
| Present-Fatalistic | 1.34_d_ | 0.27_c_ | -0.31_b_ | -0.99_a_ | -0.18_b_ | .48 |
| Positive Time Use: Total | -0.64_a_ | -0.34_a_ | 0.21_b_ | 0.43_b_ | 0.50_b_ | .18 |
| Self-congruence | -0.54_a_ | -0.34_a_ | 0.14_b_ | 0.40_b_ | 0.52_b_ | .15 |
| Balance | -0.23_a_ | -0.30_a_ | 0.23_b_ | 0.00_a_ | 0.52_b_ | .09 |
| Efficiency | -0.69_a_ | -0.13_b_ | 0.05_b_ | 0.57_c_ | 0.14_b_ | .14 |
| Mastery | -0.48_a_ | -0.31_a_ | 0.24_b_ | 0.31_b_ | 0.39_b_ | .12 |
| Time Management: Total | -0.80_a_ | 0.08_b_ | -0.23_b_ | 0.71_c_ | 0.00_b_ | .20 |
| Mechanics | -0.58_a_ | 0.10_b_ | -0.20_ab_ | 0.48_c_ | 0.00_b_ | .10 |
| Setting Goals and Priorities | -0.64_a_ | 0.07_b_ | -0.24_ab_ | 0.58_c_ | 0.05_b_ | .13 |
| Preference for Organisation | -0.85_a_ | -0.01_b_ | -0.12_b_ | 0.79_c_ | 0.00_b_ | .22 |
| Structured Routine | -0.50_a_ | 0.10_bc_ | -0.18_ab_ | 0.43_c_ | -0.04_b_ | .08 |
| Subjective Well-Being: Total | -0.49_a_ | -0.38_a_ | 0.19_b_ | 0.33_b_ | 0.55_b_ | .16 |
| Satisfaction with Life | -0.31_a_ | -0.37_a_ | 0.07_ab_ | 0.34_bc_ | 0.53_c_ | .13 |
| Positive Affect | -0.06_ab_ | -0.31_a_ | 0.03_abc_ | 0.16_bc_ | 0.43_c_ | .07 |
| Negative Affect | 0.67_b_ | 0.14_a_ | -0.30_a_ | -0.22_a_ | -0.24_a_ | .11 |

*Note.* All the omnibus differences are significant at p < .001. The subscripts denote homogeneous subsets based on Tukey post hoc test (means sharing the same subscript do not differ significantly from each other).

We interpreted the TP profiles in line with their descriptions in previous work (Zimbardo and Boyd, 2008; Boniwell et al., 2010). The first group was characterized by high scores on the Past-Negative, Present-Hedonistic, and Present-Fatalistic dimensions and was interpreted as Risk-Taking or present-oriented profile. The second group showed fairly high scores on the Past-Negative dimension combined with close-to-average scores on the other dimensions and was interpreted as Negative profile. The third group showed low scores on the Past-Negative and Past-Positive dimensions combined with close-to-average scores on the other three ZTPI scales and was interpreted as Diffuse. The fourth group showed high Future scores combined with above-average Past Positive and low Present-Hedonistic, Present-Fatalistic, and Past-Negative. This group was labelled as Future-oriented. The final, fifth profile, revealed high Present-Hedonistic and Past-Positive scores combined with low Past-Negative and close-to-average Present-Fatalistic and Future. It was labelled as Balanced, being the closest to the balanced time perspective profile, based on the Deviation from Balanced Time Perspective index.

In terms of positive time use, the Future-Oriented and Balanced groups showed comparable total scores, however, participants with the Future-Oriented time perspective profile revealed higher efficiency of time use and lower balance of activities. The Future-Oriented group reported the highest use of time management strategies and tool, whereas the Balanced group only had an average (and significantly lower) score. These two groups were also comparable in terms of well-being. In terms of time perspective, they differ on the Future and the two present scales: the Balanced group emerges as more oriented towards the present.

The Risk-taking group revealed an inverse picture of temporal ill-being: they had the lowest scores on all the positive time use dimensions, as well as time management and well-being. Finally, the groups labelled as Negative and Diffuse had comparable scores on time management and well-being scales, but the Diffuse group showed higher scores on most positive time use dimensions and the subjective well-being index. The Negative group had higher scores on both past scales and Present-Fatalistic, suggesting a focus on the past and lower openness towards the present, despite a somewhat stronger focus on the future, compare to the Diffuse group.

Thus, the findings of person-oriented approach reveal two distinct patterns of temporal well-being, Balanced and Future-oriented: despite different extent of focus on the future vs. the present they report comparable time use satisfaction and general well-being. The two patterns of temporal ill-being, Risk-Taking and Negative profiles, are characterized by the combination of a negative past and a fatalistic present orientation and also report comparably low levels of satisfaction with time use and general well-being. Future research could replicate these findings in larger samples and investigate the implications of different time perspective profiles for positive time use in a longer-term perspective.

**References**

Boniwell, I., Osin, E., Alex Linley, P., & Ivanchenko, G. V. (2010). A question of balance: Time perspective and well-being in British and Russian samples. *The Journal of Positive Psychology, 5*(1), 24-40.

Brown, T. A. (2015). *Confirmatory Factor Analysis for Applied Research. 2^nd^ Ed*. Guilford Press.

Jöreskog, K. G. (1971). Statistical analysis of sets of congeneric tests. *Psychometrika, 36*, 109–133.

Zimbardo, P., & Boyd, J. (2008). *The time paradox: The new psychology of time that will change your life*. Simon and Schuster.
